# Supplementary material for: The legacy of the extinct Neotropical megafauna on plants and biomes
Source: Nat Commun. 2022 Jan 10;13:129. doi: 10.1038/s41467-021-27749-9 (PMC8748933; doi:10.1038/s41467-021-27749-9)
Supplement: Supplementary file 1 — Supplementary Information [file 41467_2021_27749_MOESM1_ESM.pdf]

SUPPLEMENTARY INFORMATION

TITLE: The legacy of the extinct Neotropical megafauna on plants and biomes

Authors: Vinicius L. Dantas[1,*], Juli G. Pausas[2]

Affiliations:

[1] Institute of Geography, Federal University of Uberlandia (UFU), Av. João Naves de Avila, 2121, 38400-902, Uberlandia, MG, Brazil.

[2] Centro de Investigaciones sobre Desertificación, Spanish National Research Council (CIDE-CSIC), Ctra. Naquera Km. 4.5 (IVIA), Montcada, 46113, Valencia, Spain.

* Correspondence to: viniciusldantas@gmail.com

1. METHODS

A complete list of the references used to obtain trait and megafauna data (those that are not directed mentioned and cited in the main text) can be found in the References list of this Supplementary Materials and Methods. References used for stem spinescence in plant stems and branches are listed from 1 to 44, for megafauna species diet, from 45 and 65, and for latex, are 1 and from 66 to 73.

From Species to Ecoregion

As mentioned in the main text, we found occurrence GBIF data for 2110 species for wood density, 2133 for leaf size, 2629 for stem spines, 2714 for latex and 657 for leaf spines. To have an overall idea of how representative our trait data were in relation

to ecoregion patterns for the Neotropical region, we used data from Kier et al. [74], an assessment of vascular plant diversity in global ecoregions. We compared species richness in 135-136 ecoregions (from a total of 179) because the ecoregion classification map was recently modified by Dinerstein et al. [75] and because we did not find trait data for some ecoregions. Overall, there was a strong positive correlation between plant species richness in ours and in the Kier et al. dataset (r = 0.67-0.75, depending on the trait). We found that, on average (across ecoregions), the richness of the assemblages in our data represented 11-14 % of that in Kier et al. (latex: 14 %; stem spines: 12%; wood density: 11%; and leaf size: 11%; here, excluding leaf spines). However, data from [75] indicate that woody species, which is the only group considered in our study, account for only 39 % of the vascular flora in the Americas. We therefore reduced species richness in Kier[74] et al. to 39 % of that reported, resulting that, our data accounts, on average, to approximately 28-36 % of the woody flora in this study (again, depending on the trait and excluding leaf spines). In this later scenario, only 3-4 ecoregion were represented by less than 3% of the flora. In relation to the Neotropical region, estimates from [77], suggest that the total flora of the region comprises 115,242 species, for which 39% represents 44,944 species. Considering the species for which we found GBIF data (2110-2714 species; excluding Leaf Spines, for which we used palm data), our results are based on 5-6 % of the woody plant flora. The proportion of species in our leaf spines data was obviously smaller (as it only included palms) but, even for this trait, there was a strong positive correlation between species richness in our dataset and that in Kier et al. [74] (r = 0.75), indicating that this data is representative of the overall pattern.

Historical Megafauna Distribution

Different authors adopt different body mass thresholds to define what is called "megafauna". The choice of these values is, however, arbitrary, rather than biologically oriented[78]. Here, we selected the 50 kg threshold because it is close to values often used (44-45 kg[78]) but, at the same time, sounded more parsimonious in the light of the fact that body mass actually varies several orders of magnitude, reaching several thousands of kg (e.g., African savanna elephants can reach 4,000 kg). To make sure that this choice did not influence our results, we also calculated megafauna richness across ecoregions using the 40, 44 and 60 kg thresholds. We, then, evaluated how these two indices were correlated with our 50 kg-based richness index across ecoregions. In the first two cases (40 and 44 kg), the Pearson correlation coefficient at two decimal digits was equal to unity ($r = 1.00$), whereas in the third (60 kg) it was 0.99, indicating that this choice did not affect our results. We did not consider larger thresholds, such as 1,000 kg, because it is well-known that mid-size mammal herbivores have important effects in vegetation structure and function (see, e.g., [79]).

Because the effects of megafauna are commonly evaluated using animal density (generally in $Kg.Km^{-2}$ [79]), rather than richness, we also calculated megafauna density and megafauna secondary productivity using indices based on allometric equations that are commonly used by paleoecologists [80]. Density was calculated as $\log(\text{density}) = -0.75*\log(\text{Body Mass}) + 4.23$, whereas secondary productivity was calculated as $\log(\text{productivity}) = 0.67*\log(M_s) + \log(\text{density}) + 1.14$, where M_s is the equivalent in kcal of the animal's body mass, calculated by multiplying body mass (in grams) by 1.5 (the caloric value of the mammalian body in $kcal.g^{-1}$). Based on this equation, we obtained a density and a secondary productivity value for each megafauna species and multiplied the megafauna species per grid cell presence/absence matrix with the corresponding value for each species. The row (grid cell) sums were then used to

calculate the grid cell density and secondary productivity, and, then, the ecoregion scale mean. These two metrics were strongly positively correlated to megafauna richness (Supplementary Fig. 3). Since they are also based on body mass, we preferred using richness and body mass directly, as this later variable could capture additional dimensions of the effect of megafauna body mass on plants. Moreover, density is likely to be influenced by the ecosystem carrying capacity, as driven by soil and climate, an aspect that is not taken into account in these indices.

Most of the diet information used for megafauna species was based on δ13C isotopic signatures from tooths and bones placing species along a C3-C4-dominated diet spectrum. In these cases, species were classified by paleoecologists according to the prevailing diet, as either grazers (C4 plant-dominated diet) or browsers (C3 plant-dominated diet), except for species clearly showing intermediate δ13C values (classified as mixed-feeder). In other cases, diet classification was derived from morphological proxies (e.g., tooths, muzzles) and enamel wear patterns instead, indicating prevalence of grasses or browse in the diet, or a mixed diet. One of the sources was a large compilation, mostly based on δ13C signatures, in which diet information was provided as percentage of browse and graze in diets, which we directly used for classification purposes. In these cases, we classified a species as mixed-feeder only if the difference was smaller than 10%. Finally, we also classified as mixed-feeder those species found to be grazer in one site and browser in another. While these proxies often do not allow a detailed discrimination of guilds (e.g., frugivores, folivores), evidence from African mammal herbivores indicate that animals over 50 kg rarely rely on fruits and seeds as their main food resource (i.e., most use leaves and/or stems [81]). Therefore, our megafauna richness and body mass metrics were calculated under the assumption that all extinct megafauna herbivorous in the dataset fed primarily on leaves and/or stems,

even those for which no detailed dietary data (i.e., beyond strict herbivory) was available.

Biome Shifts

To validate the areas hypothesized to have experienced savanna-to-forest shifts, we compiled pollen fossil data from sites showing evidence of a past savanna state in Last Glacial Maximum, Mid-Holocene or in both periods. We found data of this type for 22 fossil sites from the literature sources listed from 82-101. Details on this data are provided in Table S10.

Statistical Analyses

To confirm the significance of all coefficients associated with megafauna and extant herbivore indicators in the selected regression models, we randomly reshuffled the plant species abundances per ecoregion matrix for all traits (for leaf spines, the species presence-absence matrix; always preserving ecoregions' totals), calculated the mean trait per ecoregion and then repeated the regressions using the same predictor variables selected in the best model for each trait, but using the randomized trait values as response. We repeated this procedure 1,000 times (one for each randomization), calculating, in each run, the standardized slope, to generate a distribution with 1,000 slopes. The 0.05 and 0.95 quantiles of the resulting distribution were compared with the observed standardized slopes for each trait and herbivory (extinct and extant) indicator that were previously found to be significantly related to each trait. To confirm these association, the observed slopes should not fall within the 0.05-0.95 quantile range of the random slope distribution.

2. FIGURES

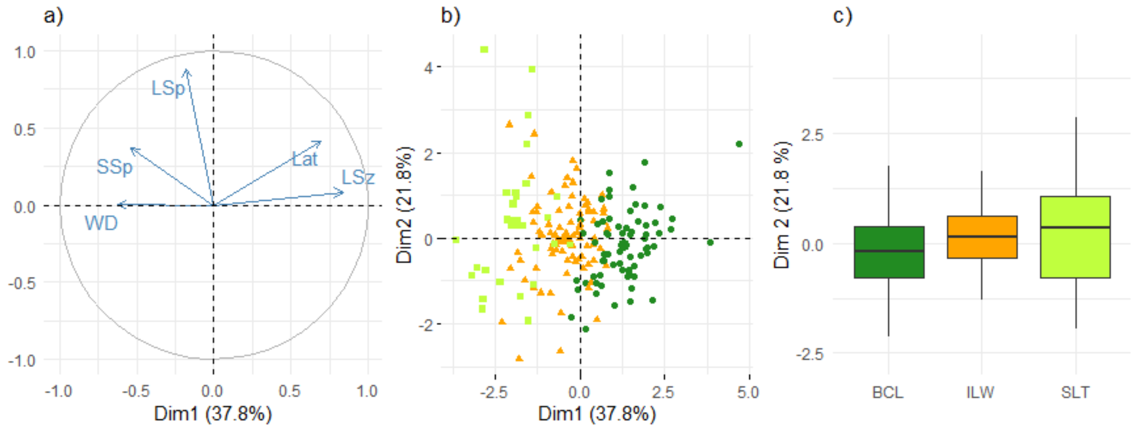

Supplementary Fig. 1: Principal Component Analyses results complementing those presented in Fig. 3. Here, patterns associated with the second axis (Dim2) are shown in detail. This axis, which was mostly related to leaf spines, did not significantly differed among antiherbiomes and, thus, was not further analysed. (a) trait axes correlation; (b) ecoregion scores; (c) antiherbiome differences in axis 2 scores (n = 150 biologically independent ecoregions). Boxplot description: center line, median; box limits, first and third quartiles; whiskers, 1.5x interquartile range; outliers not shown.

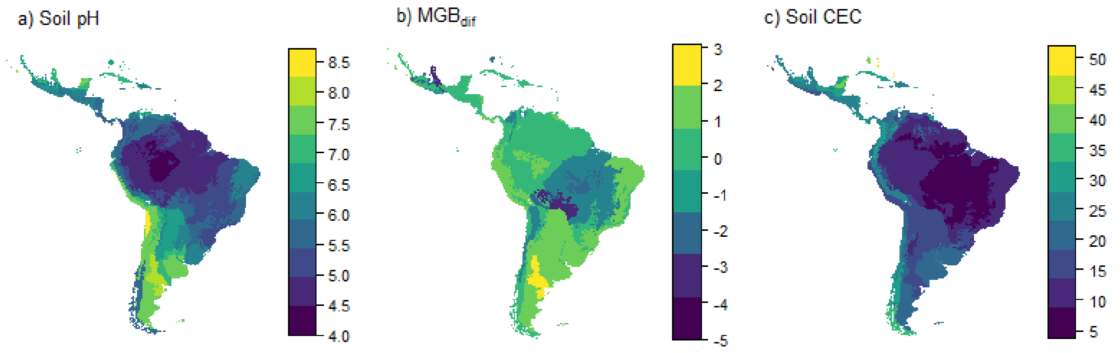

Supplementary Fig 2: Geographical variability in the most important predictors of the Physical-Chemical and the Thorny-Woody principal component axes of antiherbivory defence traits (see Figs. 3 and 5). Megafauna richness is omitted because the map of this variable was already presented in Fig. 1a. Physical defences were favoured by high soil pH (a) and a high richness of megafauna species, especially grazers (high MGB_{dif}; b), and low CEC (c), whereas chemical defences were favoured under the opposing condition. Allocation to wood density (Woody) was favoured by an even higher richness of megafauna species, whereas allocation to stem spines were favoured by extremely high soil pH. MGB_{dif}: differences between the number of megafauna grazers and browsers. More details in Fig. 5.

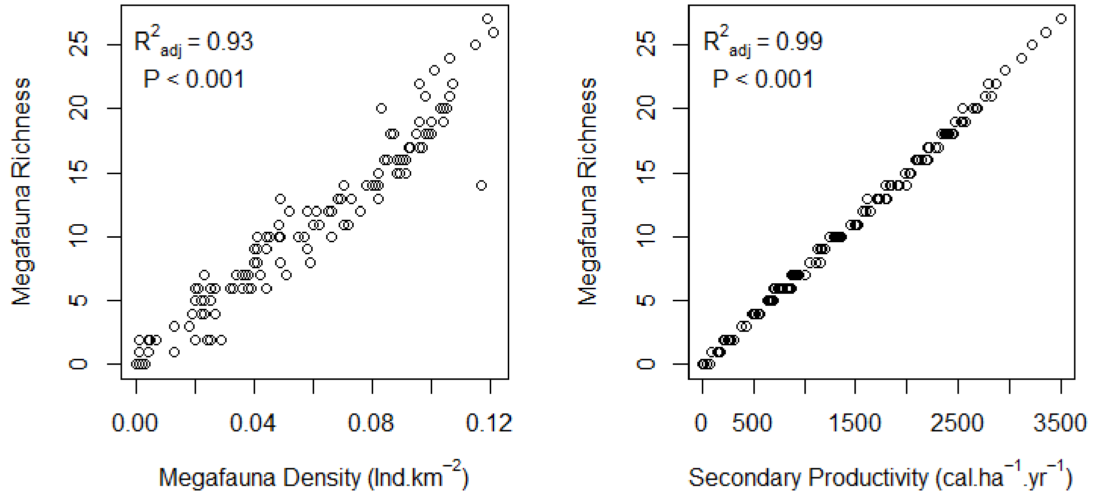

Supplementary Fig. 3: Relationship of extinct megafauna species richness with megafauna density (left) and secondary productivity (right). The latter two variables were estimated using allometric equations provided by [80]. Density was calculated as log(density) = − 0.75*log (Body Mass) + 4.23, whereas secondary productivity was calculated as log(productivity) = 0.67*log(M_s) + log(density) + 1.14, where M_s is the equivalent in kcal of the animal's body mass, calculated by multiplying body mass (in grams) by 1.5 (the caloric value of the mammalian body in kcal*g^{-1}). See [80] for more details. n = 179 biologically independent samples.

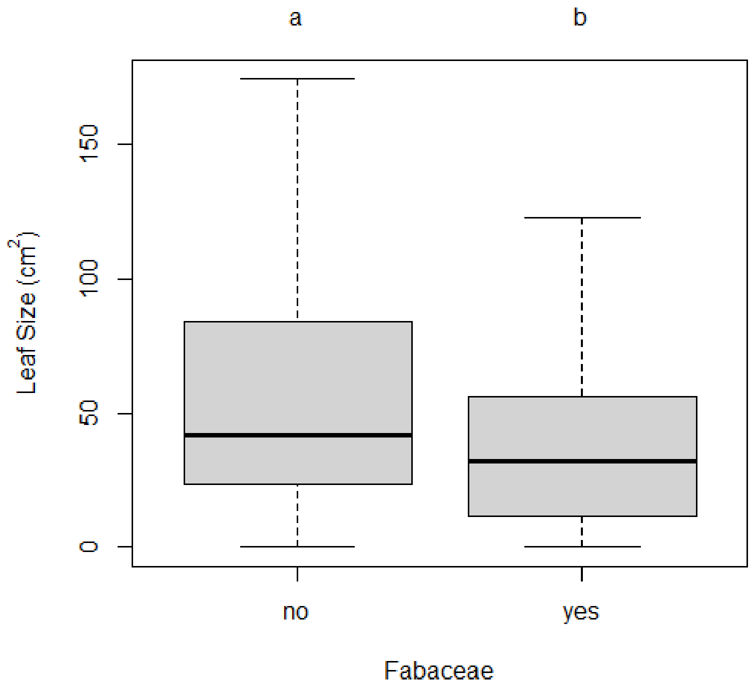

Supplementary Fig 4: Leaf size in Fabaceae vs. non-Fabaceae species in our dataset. Different letter in the top of the boxes indicate significant differences between groups in a Kruskal-Wallis test (Kruskal-Wallis χ^2 = 67.076; P = 0.000; n = 4252 biologically independent individual plants). Boxplot description: center line, median; box limits, first and third quartiles; whiskers, 1.5x interquartile range; outliers not shown.

3. Tables

Supplementary Table 1: Pairwise correlations between all pairs of candidate variables to be included as predictors in the regression models (Table S2-S4)

	M_{rich}	M_{bm}	MGB_{dif}	MG_{rich}	MB_{rich}	MMf_{rich}	H_{rich}	H_{bm}	HGB_{dif}	pH	INS	FF	FI	MAR	MAT	RS	CEC	SND	HUR
M_{rich}	1.00																		
M_{bm}	-0.44	1.00																	
MGB_{dif}	-0.11	0.07	1.00																
MG_{rich}	**0.89**	-0.30	0.15	1.00															
MB_{rich}	**0.83**	-0.30	-0.51	**0.74**	1.00														
MMf_{rich}	**0.92**	-0.37	-0.08	**0.71**	**0.64**	1.00													
H_{rich}	0.59	-0.23	-0.05	0.50	0.46	0.58	1.00												
H_{bm}	-0.15	0.44	0.05	0.06	0.07	0.27	-0.22	1.00											
HGB_{dif}	0.40	0.02	-0.25	0.44	0.54	0.26	0.15	0.27	1.00										
pH	-0.37	0.27	0.13	-0.12	-0.15	-0.55	-0.46	0.47	0.14	1.00									
INS	-0.32	-0.35	0.02	-0.33	-0.31	-0.34	-0.47	-0.26	-0.28	0.16	1.00								

FF	0.40	-0.08	-0.25	0.30	0.40	0.39	0.06	0.02	0.16	-0.04	-0.11	1.00							
FI	0.21	-0.11	-0.11	0.29	0.30	0.05	0.09	0.15	0.25	0.09	-0.15	0.22	1.00						
MAR	0.04	-0.12	-0.11	-0.17	-0.11	0.25	0.17	-0.38	-0.33	**-0.78**	0.05	-0.11	-0.23	1.00					
MAT	0.14	-0.13	-0.12	-0.07	-0.04	0.33	-0.07	-0.37	-0.29	-0.37	0.17	0.22	-0.36	0.48	1.00				
RS	-0.22	0.43	-0.08	-0.16	-0.07	-0.24	-0.28	0.30	0.26	0.43	-0.13	0.16	-0.07	-0.49	-0.03	1.00			
CEC	-0.58	0.19	-0.03	-0.49	-0.36	-0.59	-0.48	0.29	-0.15	0.42	0.39	-0.25	-0.13	-0.07	-0.19	-0.03	1.00		
SND	0.18	-0.04	0.09	0.23	0.15	0.10	0.11	0.11	0.09	0.16	-0.26	0.24	0.38	-0.31	-0.39	0.06	-0.39	1.00	
HUR	-0.34	0.20	0.06	-0.24	-0.24	-0.35	-0.47	0.29	0.06	0.41	0.29	-0.04	-0.22	-0.22	0.08	0.34	0.29	-0.21	1.00

Correlations were used to evaluate potential multicollinearities before running the regression models. Variables that showed a correlation coefficient of 0.60 or higher (bold) were not included in the same initial set of candidate variables. This occurred, for example, with extinct megafauna indicators, such as: megafauna richness (M_{rich}), megafauna browsers (MB_{rich}), grazers (MG_{rich}) and mixed-feeders (MMf_{rich}). Therefore, only megafauna richness, body mass (M_{bm}) and the difference between the number of megafauna grazers and browsers (MGB_{dif}), were included as predictors in the same trait model, whereas the others were not evaluated. In the case of mean annual rainfall and pH, since both variables were considered to be important, model selection was run separately for them and the final model with the lowest AIC was selected. H_{rich}: mean herbivore mammal species richness; H_{bm}: mean herbivore mammal species body mass; HGB_{dif}: difference between number of

mammal herbivore grazer and browser species; MAR: mean annual rainfall; MAT: mean annual temperature; RS: rainfall seasonality; SND: soil sand content; CEC: soil cation exchange capacity; pH: soil pH; FI: fire intensity; FF: fire frequency; HU: hurricanes; INS: insularity.

Supplementary Table 2: General and generalized linear model results for analyses regressing megafauna indicators, plant functional traits and principal component axes of plant functional traits (response variables) against biotic (i.e., megafauna and extant mammal species; for traits and PCA axis) and abiotic (climate, soil, fire and hurricanes) predictors.

	**Response variables**	***N***	**df**	**R^2_{adj}**	**R^2_{McFad}**	**R^2_{ML}**	**R^2_{CU}**	**ΔAIC**
Megafauna	M_{rich} (number)	157	150	0.53				-114.02
	Log (M_{bm} (index) + 1)	157	152	0.56				-123.12
	Log (MB_{rich} (number) + 1)	157	151	0.39				-72.01
	MG_{rich} (number)	157	150	0.43				-83.79
	MMf_{rich} (number)	157	150	0.57				-128.28
Plants	Wood Density ($g.cm^3$)	143	137	0.47				-85.38
	Stem Spines (yes/no)	143	134		0.53	0.69	0.78	-151.76
	Leaf Size (cm^2)	143	137	0.59				-121.21
	Leaf Spines (yes/no)	132	128		0.15	0.38	0.40	-345.61
	Latex (yes/no)	143	139		0.19	0.25	0.33	-32.52
	Physical-Chemical (Dim1)	143	138	0.57				-117.25
	Thorny-Woody (Dim3)	143	139	0.42				-74.11

Results are for the best stepwise selected model (based on AIC; models are shown in detail in Table S3; model residual diagnostics are shown in Table S4). All models are multiple general linear regressions except for situations where stem and leaf spines, and latex were the response variables, in which case the results are from generalized linear model with a binomial family distribution. For the latter, MacFadden's ($R2_{McFad}$), maximum likelihood (R^2_{ML}), and Cragg and Uhler's (R^2_{CU}) pseudo-R^2 are shown instead of the adjusted R^2 (R^2_{adj}). ΔAIC: Change in Akaike Information Criterium (difference between selected and null model). *N*: number of observations (ecoregions); M_{rich}: mean

extinct megafauna species richness; M_{bm}: mean extinct megafauna species mean body mass; MG_{rich}, MB_{rich} and MMf_{rich} (C and D): mean extinct megagrazer, megabrowser and megamixed-feeders species richness, respectively; Physical-Chemical and Thorny-Woody: first and third principal component axes of plant functional traits per ecoregion (only axes that significantly differ among antiherbiomes were evaluated; see Fig. 3 and S2 for more details).

Supplementary Table 3: Selected regression models and detailed statistical results for the models summarized in Supplementary Table 2.

		r	95% CI (*r*)	Av. Contr. (R^2)	Coef.	SE	95% CI (Coef.)	*t*|*z**	*P*
M_{rich}	(Intercept)	-	-	-	34.32	3.87	[26.68, 41.96]	8.87	0.000
	INSUL	-0.22	[-0.36, -0.06]	0.06	-3.69	1.33	[-6.32, -1.06]	-2.77	0.006
	MAR	-0.25	[-0.38, -0.09]	0.01	0.00	0.00	[0.00, 0.00]	-3.12	0.002
	RS	-0.45	[-0.56, -0.32]	0.08	-0.10	0.02	[-0.13, -0.07]	-6.23	0.000
	CEC	-0.56	[-0.65, -0.45]	0.27	-0.42	0.05	[-0.53, -0.32]	-8.34	0.000
	SND	-0.24	[-0.38, -0.09]	0.02	-0.17	0.06	[-0.29, -0.06]	-3.06	0.003
	FF	0.42	[0.29, 0.54]	0.12	0.00	0.00	[0.00, 0.01]	5.75	0.000
M_{bm}	Intercept	-	-	-	13.01	0.25	[12.53, 13.49]	53.08	0.000
	INSUL	-0.71	[-0.77, -0.64]	0.45	-2.08	0.17	[-2.41, -1.76]	-12.57	0.000
	MAR	0.25	[0.09, 0.39]	0.01	0.00	0.00	[0.00, 0.00]	3.16	0.002
	RS	0.37	[0.23, 0.49]	0.08	0.01	0.00	[0.01, 0.01]	4.99	0.000
	CEC	0.32	[0.17, 0.44]	0.03	0.02	0.01	[0.01, 0.04]	4.11	0.000
MG_{rich}	(Intercept)	-	-	-	6.57	0.55	[5.48, 7.65]	11.94	0.000

	INSUL	-0.22	[-0.36, -0.07]	0.07	-1.02	0.36	[-1.73, -0.30]	-2.81	0.006
	MAR	-0.39	[-0.51, -0.25]	0.06	0.00	0.00	[0.00, 0.00]	-5.23	0.000
	RS	-0.43	[-0.54, -0.29]	0.07	-0.03	0.00	[-0.03, -0.02]	-5.77	0.000
	CEC	-0.44	[-0.55, -0.31]	0.19	-0.08	0.01	[-0.11, -0.05]	-6.06	0.000
	FF	0.25	[0.09, 0.39]	0.06	0.00	0.00	[0.00, 0.00]	3.15	0.002
MB_{rich}	(Intercept)	-	-	-	2.73	0.41	[1.91, 3.55]	6.59	0.000
	INSUL	-0.36	[-0.48, -0.21]	0.12	-0.67	0.14	[-0.95, -0.39]	-4.68	0.000
	MAR	-0.26	[-0.39, -0.10]	0.02	0.00	0.00	[0.00, 0.00]	-3.27	0.000
	RS	-0.32	[-0.45, -0.17]	0.04	-0.01	0.00	[-0.01, 0.00]	-4.18	0.000
	CEC	-0.34	[-0.47, -0.19]	0.13	-0.02	0.01	[-0.04, -0.01]	-4.45	0.000
	SND	-0.17	[-0.31, -0.01]	0.01	-0.01	0.01	[-0.02, 0.00]	-2.07	0.040
	FF	0.35	[0.20, 0.48]	0.10	0.00	0.00	[0.00, 0.00]	4.59	0.000
MM_{rich}	(Intercept)	-	-	-	10.53	1.67	[7.24, 13.83]	6.31	0.000
	INSUL	-0.27	[-0.40, -0.11]	0.07	-2.13	0.63	[-3.38, -0.89]	-3.39	0.000
	MAT	0.24	[0.08, 0.37]	0.06	0.10	0.03	[0.03, 0.17]	2.96	0.004

	pH	-0.23	[-0.37, -0.08]	0.14	-0.07	0.02	[-0.12, -0.02]	-2.92	0.004
	RS	-0.28	[-0.41, -0.12]	0.05	-0.03	0.01	[-0.04, -0.01]	-3.53	0.000
	CEC	-0.39	[-0.50, -0.24]	0.18	-0.12	0.02	[-0.17, -0.07]	-5.12	0.000
	FF	0.36	[0.22, 0.49]	0.10	0.00	0.00	[0.00, 0.00]	4.78	0.000
WD	(Intercept)	-	-	-	0.36	0.03	[0.30, 0.43]	10.87	0.000
	M_{rich}	0.56	[0.44, 0.65]	0.26	0.01	0.00	[0.00, 0.01]	7.93	0.000
	MGB_{dif}	0.32	[0.16, 0.45]	0.05	0.01	0.00	[0.01, 0.02]	3.91	0.000
	MAT	0.28	[0.12, 0.42]	0.06	0.00	0.00	[0.00, 0.00]	3.43	0.000
	HUR	0.35	[0.20, 0.48]	0.05	0.00	0.00	[0.00, 0.01]	4.44	0.000
	SND	0.37	[0.22, 0.50]	0.08	0.00	0.00	[0.00, 0.00]	4.69	0.001
SSp	(Intercept)	-	-	-	-9.74	0.84	[-11.41, -8.10]	-11.55	0.000
	M_{bm}	0.21	[0.05, 0.36]	0.02	0.00	0.00	[0.00, 0.00]	2.61	0.010
	M_{bm}^2	-0.19	[-0.33, -0.03]	0.02	0.00	0.00	[-0.00, 0.00]	-2.27	0.020
	MGB_{dif}	0.20	[0.04, 0.35]	0.02	0.11	0.04	[0.02, 0.20]	2.50	0.010
	Hrich	0.29	[0.14, 0.42]	0.02	0.05	0.01	[0.02, 0.08]	3.64	0.000

	HGB_{dif}	0.24	[0.08, 0.38]	0.05	0.16	0.05	[0.05, 0.27]	2.97	0.000
	FI	0.17	[0.00, 0.32]	0.02	0.00	0.00	[0.00, 0.00]	2.01	0.040
	MAT	0.36	[0.22, 0.48]	0.03	0.06	0.01	[0.03, 0.08]	4.60	0.000
	pH	0.64	[0.55, 0.70]	0.34	0.07	0.01	[0.06, 0.09]	9.83	0.000
LSp	(Intercept)	-	-	-	-2.21	0.38	[-2.96, -1.48]	-5.86	0.000
	M_{rich}	0.46	[0.33, 0.57]	0.09	0.05	0.01	[0.03, 0.07]	5.95	0.000
	RS	-0.25	[-0.39, -0.09]	0.02	-0.01	0.00	[-0.01, 0.00]	-2.95	0.000
	MAT	0.30	[0.15, 0.44]	0.04	0.05	0.01	[0.02, 0.08]	3.66	0.000
LSz	(Intercept)	-	-	-	165.06	16.81	[131.82, 198.30]	9.82	0.000
	M_{rich}	-0.53	[-0.63, -0.41]	0.11	-1.73	0.23	[-2.19, -1.27]	-7.37	0.000
	MGB_{dif}	-0.21	[-0.36, -0.04]	0.03	-3.01	1.22	[-5.42, -0.61]	-2.48	0.020
	MAT	0.34	[0.18, 0.47]	0.12	1.20	0.29	[0.63, 1.77]	4.19	0.000
	pH	-0.60	[-0.69, -0.49]	0.26	-1.56	0.18	[-1.91, -1.22]	-8.86	0.000
	SND	-0.20	[-0.35, -0.03]	0.08	-0.48	0.20	[-0.88, -0.08]	-2.38	0.020
Lat	(Intercept)	-	-	-	-1.25	0.31	[-1.85, -0.64]	-4.04	0.000

	H_{bm}	-0.32	[-0.45, -0.17]	0.10	0.00	0.00	[0.00, 0.00]	-3.99	0.000
	CEC	0.32	[0.17, 0.45]	0.04	0.02	0.01	[0.01, 0.04]	4.03	0.000
	pH	-0.17	[-0.32, -0.01]	0.05	-0.01	0.01	[-0.02, 0.00]	-2.07	0.040
P-C	(Intercept)				7.63	0.65	[6.34, 8.92]	11.72	0.000
	M_{rich}	-0.49	[-0.60, -0.36]	0.13	-0.10	0.02	[-0.13, -0.07]	-6.68	0.000
	MGB_{dif}	-0.24	[-0.38, -0.07]	0.04	-0.21	0.07	[-0.35, -0.06]	-2.87	0.010
	pH	-0.72	[-0.78, -0.63]	0.38	-0.12	0.01	[-0.14, -0.10]	-12.05	0.000
	CEC	0.22	[0.06, 0.37]	0.04	0.03	0.01	[0.01, 0.06]	2.67	0.010
T-W	(Intercept)				1.83	0.55	[0.75, 2.92]	3.35	0.000
	M_{rich}	0.41	[0.27, 0.53]	0.17	0.06	0.01	[0.04, 0.09]	5.33	0.000
	HGB_{dif}	-0.27	[-0.41, -0.11]	0.05	-0.23	0.07	[-0.37, -0.09]	-3.27	0.000
	pH	-0.40	[-0.52, -0.26]	0.22	-0.04	0.01	[-0.06, -0.03]	-5.20	0.000

* *z* statistics for stem spines (SSP), leaf spines (LSP) and latex (LAT) (i.e., for binary plant traits), and *t* for other response variables (i.e., the continuous response variables). Models were selected based in the lowest AIC, after which non-significant variables were stepwise deleted until all predictor variables had a significant independent effect. Observations are ecoregions of the Neotropical realm (see more information in Table

S2). r: Pearson correlation statistics; Av. Contr. (R^2): average variable contribution in terms of R^2 (MacFadden Psedo-R^2 for binary and R^2 for continuous traits); CI (r) and CI (Coef.): confidence interval for r and for coefficients, respectively; SE: standard error; M_{rich}: mean extinct megafauna species richness; M_{bm}: mean extinct megafauna species body mass; MG_{rich}, MB_{rich} and MMf_{rich}: mean extinct megagrazer, megabrowser and megamixed-feeders species richness, respectively; MGB_{dif}: difference between mean number of megafauna grazers and browsers; H_{rich}: mean pre-historical richness of extant mammal herbivores; HGB_{dif}: difference between the mean pre-historical richness of grazers and browsers; H_{bm}: mean pre-historical body mass of extant mammal herbivores; WD: wood density; SSp: Stem spines; LSp: leaf spines; LSz: Leaf Size; Lat: Latex; MAT: mean annual temperature; MAR: mean annual rainfall; RS: rainfall seasonality; SND: soil sand content; pH: soil pH; CEC: soil cation exchange capacity; FI: (wild)fire intensity; FF: (wild)fire frequency; HUR: hurricane activity (count per area); P-C and W-T: First (named Physical-Chemical) and third (named Thorny-Woody) principal component axes of plant functional traits (only axes that significantly differ among antiherbiomes were evaluated; see Fig. 3 and S1 for more details on these axes).

Supplementary Table 4: Residual diagnostics for the regression models presented in Supplementary Tables 2 and 3

	Kolmogorov-Smirnov		Breusch-Pagan Test		HCE	Dispersal		Spatial Autocorrelation	
Response	Statistic	*P*	Statistic	*P*	Robust predictors	Residual Dev	DF	Moran I SSD	*P*
M_{rich}	0.10	0.107	16.985	0.009	all	-	-	0.129	0.449
M_{bm}	0.10	0.117	79.441	0.000	all	-	-	1.040	0.149
MG_{rich}	0.09	0.167	26.924	0.000	All but one (removed)	-	-	0.295	0.384
MB_{rich}	0.06	0.662	10.101	0.121	-	-	-	-0.675	0.750
MM_{rich}	0.05	0.912	20.697	0.002	all	-	-	0.580	0.281
WD	0.06	0.632	4.927	0.425	-	-	-	0.748	0.227
LSz	0.08	0.328	40.822	0.000	all	-	-	-1.121	0.869
SSp	-	-	-	-	-	119.63	134	0.812	0.208
LSp	-	-	-	-	-	123.10	128	0.761	0.224
Lat	-	-	-	-	-	138.68	139	0.890	0.187
P-C	0.07	0.543	21.531	0.002	All but two (removed)	-	-	-0.736	0.769

T-W	0.07	0.590	1.385	0.709	-	-	-	0.369	0.356

Kolmogorov-Smirnov, Breusch-Pagan and Moran I tests were used to test the residuals of the models presented in Table S3 against the null hypothesis of normality, homoscedasticity and no spatial autocorrelation, respectively. In none of the cases non-normality or spatial autocorrelation was detected. In some cases, heteroscesticity was detected and could not be cured through data transformation (a significant P in the Breusch-Pagan test). Thus, we used heteroskedasticity-consistent estimation (HCE) of covariance matrices to check predictor significance and, if one or more variables lost their significances, the variable was stepwise removed from the model, starting from the least significant. The number of variables in the model that remained significant using this approach is shown under the column "Robust predictors". For the logistic models (generalized linear regression with binomial error), residual deviance (Resid. Dev.) and degrees of freedoms (DF) are shown for overdispersal diagnosis. M_{rich}: mean extinct megafauna species richness; M_{bm}: mean extinct megafauna species mean body mass; MG_{rich}, MB_{rich} and MM_{rich}: mean extinct megagrazer, megabrowser and megamixed-feeders species richness, respectively. WD: wood density; LSz: leaf size; SSp: stem spines; LSp: leaf spines; Lat: latex; P-C: first PCA axis of plant traits (named Physical-Chemical); T-W: third PCA axis of plant traits (named Thorny-Woody).

Supplementary Table 5: Simulation results for the relationships between traits (response variable) and the significant herbivory predictors (in the models shown in Supplementary Table 3)

		WD	LSz	SSp	LSp	Lat
M_{rich}	Observed	0.528	-0.434		0.301	
	Simul. (5%)	-0.128	-0.202		-0.056	
	Simul. (95%)	0.126	0.183		0.059	
MGB_{dif}	Observed	0.244	-0.128	0.121		
	Simul. (5%)	-0.104	-0.123	-0.006		
	Simul. (95%)	0.092	0.121	0.006		
M_{bm}	Observed			0.772		
	Simul. (5%)			-0.046		
	Simul. (95%)			0.049		
M_{bm}^2	Observed			-0.654		
	Simul. (5%)			-0.053		
	Simul. (95%)			0.049		
H_{rich}	Observed			0.242		
	Simul. (5%)			-0.007		
	Simul. (95%)			0.008		
HGB_{dif}	Observed			0.163		
	Simul. (5%)			-0.006		
	Simul. (95%)			0.006		
H_{bm}	Observed					-0.236
	Simul. (5%)					-0.008

Simul. (95%)	0.008

Simulated trait values were obtained after 1,000 random shuffles of the species abundance per ecoregion matrix while respecting total row counts (richness, in the case of leaf spines, and total abundance for other traits). Simul. (5%) and Simul. (95%) represent, respectively, the 5 and 95 % quantiles of the distribution of standardized slopes obtained after replacing the observed ecoregion trait values by the values obtained in the 1,000 random simulations in the models shown in Supplementary Table 3. A significant association is depicted when the observed value is not included within the quantile range. WD: wood density; LSz: leaf size; SSp: stem spines; LSp: leaf spines; Lat: latex. M_{rich}: mean extinct megafauna species richness; M_{bm}: mean extinct megafauna species mean body mass; MGB_{dif}: difference between mean number of megafauna grazers and browsers; H_{rich}: mean pre-historical richness of extant mammal herbivores; HGB_{dif}: difference between the mean pre-historical richness of grazers and browsers; H_{bm}: mean pre-historical body mass of extant mammal herbivores.

Supplementary Table 6: Results of models replacing the main significant herbivory richness index (either M_{rich}, for extinct megafauna species richness, or H_{rich}, for extant mammal richness) by the richness of browsers only (either MB_{rich}, for extinct megafauna browser richness, or HB_{rich}, for extant mammal browsers richness) or by megafauna density (M_{den}; individuals per km^2; see Fig. S3).

	M_{rich}		MB_{rich}		M_{den}		H_{rich}		HB_{rich}	
	**AIC**	R^2	**AIC**	R^2	**AIC**	R^2	**AIC**	R^2	**AIC**	R^2
Wood Density ($g.cm^3$)	-485.29	0.47	-489.10	0.48	-488.17	0.48	-	-	-	-
Leaf Size (cm^2)	1202.17	0.59	1198.80	0.60	1211.19	0.56	-	-	-	-
Leaf Spines (yes/no)	367.63	0.15	359.26	0.14	365.3	0.14	-	-	-	-
Stem Spines (yes/no)	-	-	-	-	-	-	167.01	0.53	167.08	0.48
Physical-Chemical	389.24	0.57	396.39	0.55	394.87	0.55	-	-	-	-
Thorny-Woody	326.79	0.43	347.52	0.33	319.85	0.44	-	-	-	-

Based on the Akaike Information Criterium (AIC), we can observe slight improves for Wood Density and Leaf Spines when M_{rich} was replaced by either MB_{rich} and M_{den}. For leaf size, the same is observed when replacing M_{rich} by MB_{rich} and, for the Thorny-Woody axis, when replacing M_{rich} by M_{den} (but not for other response variables). Physical-Chemical and Thorny-Woody are principal components axes of antiherbivory defence traits (see Fig. 3 and S1 for details).

Supplementary Table 7: Comparison of megafauna, extant herbivore, fire, climate and soil indicators among antiherbiomes (also shown in Fig 4)

	Kruskal-Wallis			Dunn Post-hoc test					
				BCL - ILW		BCL - SLT		ILW - SLT	
	N	$\chi2$	*P*	*Z*	*P*	*Z*	*P*	*Z*	*P*
M_{rich}	150	21.36	0.000	-4.60	0.000	-1.32	0.186	2.10	0.054
M_{bm}	145	29.47	0.000	4.96	0.000	-0.23	0.821	-3.95	0.000
MGB_{dif}	150	8.50	0.019	-0.44	0.663	-2.84	0.014	-2.52	0.018
MG_{rich}	150	22.20	0.000	-4.12	0.000	-3.66	0.000	-0.60	0.550
MB_{rich}	150	13.49	0.002	-3.58	0.001	-2.09	0.551	0.57	0.568
MMf_{rich}	150	32.50	0.000	-5.09	0.000	0.49	0.623	4.27	0.000
H_{bm}	145	35.29	0.000	3.09	0.002	-3.58	0.000	-5.87	0.000
H_{rich}	150	5.58	0.075						
HGB_{dif}	150	19.44	0.000	-0.82	0.411	-4.33	0.000	-3.72	0.000
FF	146	15.48	0.001	-3.66	0.000	0.00	0.999	2.74	0.009
FI	143	3.01	0.222						
CEC	146	21.62	0.000	4.58	0.000	0.91	0.363	-2.52	0.017
SND	146	3.26	0.208						
MAT	148	22.19	0.000	-2.90	0.006	2.39	0.017	4.53	0.000
MAR	148	41.31	0.000	0.61	0.545	6.17	0.000	5.69	0.000
RS	148	5.24	0.083						
pH	146	46.96	0.000	1.13	0.257	-5.88	0.000	-6.67	0.000
P-C	150	106.58	0.000	7.47	0.000	9.39	0.000	3.84	0.000
LSA	150	5.02	0.081						
T-W	150	81.88	0.000	-7.03	0.000	2.68	0.007	7.90	0.000

Results are from a Kruskal-Wallis testing for differences among antiherbiomes, followed by a pairwise post-hoc Dunn test. Antiherbiomes are: broad chemically-defended leaves (BCL); intermediate leaves woody (ILW); and small leaves thorny (SLT). LSA: Leaf Spine PCA axis. Other variables abbreviations are as in Supplementary Table 1. *N*: number of biologically independent ecoregions.

Supplementary Table 8: Relationship of leaf size with extant and recently extinct mammal richness and difference between grazer and browser richness.

	Hrich		HGBdif	
	r	*P*	*r*	*P*
LSz	0.169	0.044	-0.383	0.000

During model selection, associations between herbivory indicators and traits that were not consistent with herbivores as the cause (i.e., herbivores did not promote more defended plants) were discarded. This only happen with leaf size, for which the significant direct relationship with extant mammal metrics is shown here for clarity purposes. n = 143 biologically independent ecoregions.

Supplementary Table 9: Sources for pollen fossil data used to validate the biome shift proposition (Fig. 6 of the main text).

Site	Lat	Lon	Current Local Vegetation	Ecoregion	HOLO Vegetation	LGM Vegetation	Past Savanna Period	Shifted to forest?	Reference
Carajás	-5.00	-48.00	Semideciduous forest	Tocantins/Pindare moist forests	Savanna	Savanna	both	yes	82
Laguna El Pinal	4.13	-70.38	Savanna/Forest	Llanos		Savanna	lgm	yes	83
Laguna Loma Linda	3.30	-73.38	Forest	Llanos		Savanna	lgm	yes	84
Catas Altas	-20.08	-43.37	Semideciduous forest (and grassland)	Bahia interior forests		Subtropical grassland	lgm	yes	85
Itapoá	-26.07	-48.63	Tropical forest	Serra do Mar coastal forests	Tropical forest	Subtropical grassland	lgm	yes	86
Serra Campos Gerais	-24.66	-50.21	Mixed forest	Cerrado	Subtropical grassland (with Araucaria)		holo	yes	87
Morro de Itapeva	-22.78	-45.63	Mixed forest	Serra do Mar coastal forests	Subtropical grassland (with Araucaria)	Subtropical grassland	both	yes	88
Cambara do Sul	-29.05	-50.10	Mixed forest	Araucaria moist forests	Subtropical grassland (with Araucaria)	Subtropical grassland	both	yes	89
Porto Velho/Humaitá	-8.18	-63.52	Savanna	Purus-Madeira moist forests	Savanna	not informed	holo	no	90
Saquinho	-10.44	-43.23	Caatinga (savanna and deciduous forest)	Cerrado	Caatinga (savanna and deciduous forest)		holo	no	91
Jacareí	-23.28	-45.97	Tropical forest	Serra do Mar coastal forests	Savanna		holo	yes	92

Carajás	-6.58	-49.50	Equatorial forest (transition with savanna)	Xingu-Tocantins-Araguaia moist forests	Savanna		holo	yes	93
Laguna Bella Vista	-13.62	-61.55	Forest	Chiquitano dry forests	Savanna	Savanna	both	yes	94; 95
Laguna Chaplin	-14.47	-61.07	Forest	Chiquitano dry forests	Savanna	Savanna	both	yes	94; 95
Lagoa Santa	-19.63	-43.90	Deciduous forest	Cerrado	Savanna (vereda-forest)		holo	yes	96
Crominia	-17.28	-49.45	Deciduous forest	Cerrado	Savanna (vereda-forest)	Savanna (vereda-forest)	both	yes	97
Aguas Emendadas	-15.56	-47.58	Savanna	Cerrado	Savanna (vereda-forest)	Savanna	both	no	97
Lago Caço	-2.97	-43.42	Savanna	Maranhão Babaçu forests		Savanna	lgm	no	98
Katira	-9.00	-63.00	Deciduous forest	Madeira-Tapajós moist forests		Savanna	lgm	yes	99
Carajás	-6.58	-49.50	Equatorial forest (transition with savanna)	Xingu-Tocantins-Araguaia moist forests		Savanna	lgm	yes	99
Serra do Caparaó	-20.41	-41.83	Highland grassland	Campos Rupestres montane savanna	Highland grassland		holo	no	100
Rio Paraiba do Sul Basin	-21.62	-39.97	Mostly rainforest	Multiple		Subtropical grassland	lgm	yes	101

LGM or lgm: last glacial maximum; HOLO or holo: mid-holocene; Lat: latitude; Lon: longitude.

4. REFERENCES

1. Flora do Brasil 2020. Jardim Botânico do Rio de Janeiro. Disponível em: < http://floradobrasil.jbrj.gov.br/ >. Acesso entre junho de 2020 e junho de 2021.

2. Loto, D. & Bravo, S. Species composition, structure, and functional traits in Argentine Chaco forests under two different disturbance histories. *Ecol. Indic.* **113**, (2020).

3. Kissling, W. D. *et al.* Data from: PalmTraits 1.0, a species-level functional trait database for palms worldwide, v4. *Dryad* (2019) doi:https://doi.org/10.5061/dryad.ts45225.

4. Carvalho, P. E. R. *Juazeiro (Ziziphus joazeiro)*. *Circular Técnica - Empresa Brasleira de Pesquisa Agropecuária* 8 (2007).

5. Carneiro, J. A. A. *et al.* Flora of Ceará, Brazil: Cleomaceae. *Rodriguesia* **69**, 1659–1672 (2018).

6. Rodrigues, E. de M., Queiroz, R. T. de, Silva, L. P. da, Monteiro, F. K. da S. & Melo, J. I. M. de. Fabaceae em um afloramento rochoso no semiárido brasileiro. *Rodriguésia* **71**, 1–25 (2020).

7. Ferreira, J. J. da S., Ana Carla da Silva Oliveira Rubens Teixeira de Queiroz & Silva, J. S. A tribo Dalbergieae s.l. (Leguminosae-Papilionoideae) no município de Caetité, Bahia, Brasil. *Rodriguésia* **70**, e03502017 (2019).

8. Freire Júnior, J. M. S. & Silva, J. S. Clado Mimosoide (Leguminosae e Caesalpinioideae) no Parque Estadual da Serra dos Montes Altos, Bahia, Brasil. *Rodriguésia* **70**, e04162017 (2019).

9. Martins, E. G. A. & Pirani, J. R. Flora da serra do cipó, minas gerais: moraceae.

Bol. Botânica da Univ. São Paulo **28**, 69–86 (2010).

10. Da Silva, S. A. L. & Miranda de Melo, J. I. A família Leguminosae Juss. em dois afloramentos rochosos no município de Puxinanã, Paraíba. *Biotemas* **26**, 23–43 (2013).

11. Nepomuceno, F. Á. A. Salicaceae na mata atlântica do nordeste oriental. vol. 2 (Universidade Federal de Pernambuco, 2018).

12. Chagas, A. P., Dutra, V. F. & Garcia, F. C. P. Flora do Espírito santo: Ingeae (Leguminosae): Parte 1. *Rodriguesia* **68**, 1613–1631 (2017).

13. Martins, M. Leguminosas arbustivas e arbóreas de fragmentos florestais remanescentes no noroeste paulista, Brasil. (Universidade Estadual Paulista, 2009).

14. Lima, T. E., Sartori, A. L. B. & Rodrigues, M. L. M. Plant antiherbivore defenses in Fabaceae species of the Chaco. **77**, 299–303 (2017).

15. Santos-Silva, J., Simon, M. F. & De Azevedo Tozzi, A. M. G. Revisão taxonômica das espécies de Mimosa ser. Leiocarpae sensu lato (Leguminosae - Mimosoideae). *Rodriguesia* **66**, 95–154 (2015).

16. Borges, R. L. A família Rubiaceae na Serra Geral de Licínio de Almeida, Bahia, Brasil. (Universidade Estadual de Feira de Santana, 2016).

17. Luz, C. L. da S. Anacardiaceae R.Br. na Flora Fanerogâmica do Estado de São Paulo. (Universidade de São Paulo, 2011).

18. São-Mateus, W. M. B., Cardoso, D., Jardim, J. G. & Queiroz, L. P. De. Papilionoideae (Leguminosae) na Mata Atlântica do Rio Grande do Norte , Brasil Introdução Material e Métodos. *Biota Neotrop.* **13**, 315–362 (2013).

19. Silva, L. de A. A tribo Mimoseae Bronn. (leguminosae) no Espirito Santo. (Universidade Federal do Espírito Santo, 2016).

20. Almeida, G. S. S. De, Carvalho-okano, R. M. De & Nakajima, J. N. Asteraceae Dumort nos campos rupestres do Parque Estadual do Itacolomi, Minas Gerais, Brasil: Barnadesieae e Mutisieae. *Rodriguésia* **65**, 311–328 (2014).

21. Manoel, E. A. & Guimarães, E. F. O gênero Strychnos (Loganiaceae) no Estado do Rio de Janeiro, Brasil. *Rodriguésia* **60**, 865–877 (2009).

22. Souza, H. B. Z. de. Celtis L . (Cannabaceae) do Brasil. (Secretaria de Estado do Meio Ambiente, 2019).

23. Saka, M. N. Solanaceae na Reserva Biológica municipal da Serra do Japi, Jundiaí, SP. (Universidade Estadual Paulista, 2009).

24. Lima, J. R. & Mansano, V. de F. A família Leguminosae na Serra de Baturité, Ceará, uma área de floresta atlântica no semiárido brasileiro. *Rodriguésia* **62**, 563–613 (2011).

25. Kirmse, R. D., Pfister, J. A., Vale, L. V & Queiroz, J. S. *Woody plants of the Northern Ceará Caatinga*. *EMBRAFA/Centro Nacional de Pesquisa de Caprinos Utah State University, Department of Range Science* vol. 14 (1983).

26. Pereira, Z. V. & Kinoshita, L. S. Rubiaceae Juss. do Parque Estadual das Várzeas do Rio Ivinhema, MS, Brasil. *Hoehnea* **40**, 205–251 (2013).

27. Santos, L. B. dos. Sistemática e filogenia de Maytenus Molina (Celastraceae) na região neotropical. (Universidade Estadual Paulista, 2016). doi:10.1017/CBO9781107415324.004.

28. Hieda, S. M. A família Euphorbiaceae na Reserva Biológica Municipal da Serra

do Japi, Jundiaí, SP. (Universidade Estadual Paulista, 2012).

29. Martins, E. G. A. O Clado Urticóide (Rosales) na Flora da Serra do Cipó, Minas Gerais. (Universidade de São Paulo, 2009).

30. Silva, L. de A., Thomaz, L. D. & Dutra, V. F. Leguminosae no Parque Natural Municipal de Jacarenema, Vila Velha, Espírito Santo, Brazil. *Iheringia - Ser. Bot.* **73**, 261–289 (2018).

31. Miller, D. Z. & Blum, C. T. Dendrological key and characterization of the vegetative morphology of Fabaceae woody species in a fragment of Araucaria Rainforest, Curitiba, PR. *Rodriguesia* **69**, 787–804 (2018).

32. Zappi, D. C., Miguel, L. M., Sobrado, S. V. & Salas, R. M. Flora das cangas da Serra dos Carajás, Pará, Brasil: Rubiaceae. *Rodriguesia* **68**, 1091–1137 (2017).

33. Pessoa, M. do C. R. & Barbosa, M. R. D. V. The family Rubiaceae Juss. in the Cariri region of Paraíba. *Rodriguésia* **63**, 1019–1037 (2012).

34. Franco, I. M. Asteraceae do Parque Estadual do Biribiri, Diamantina, Minas Gerais: Barnadesieae, Mutisieae sensu lato, Astereae e Senecioneae. (Universidade Federal de Uberlândia, 2014).

35. Claros, A. F. F. Contribution to the woody flora of the lowland forests of eastern Santa Cruz; additions to the ‘Guia de arboles’ of Bolivia. *Rev. la Soc. Boliv. Bot.* **2**, 46–59 (1998).

36. Carrión, J. F., Gastauer, M., Mota, N. M. & Meira-Neto, J. A. A. Facilitation as a driver of plant assemblages in Caatinga. *J. Arid Environ.* **142**, 50–58 (2017).

37. Marcon, T. R. Levantamento de Leguminosae arbóreas do corredor de biodiversidade Santa Maria-PR e germinação de sementes de Mimosa

bimucronata (DC.) Kuntze. (Universidade Estadual do Oeste do Paraná, 2013).

38. Queiroz, R. T. de, Medeiros, J. R. de & Trejo, I. Levantamento florísticos das plantas vasculares da RPPN fazenda Santa Clara - São João do Cariri (bioma Caatinga). in *Educaçao Ambiental em Unidades de Conservaçao no Bioma Caatinga: Biodiversidade e Formação Continuada de Professores tendo como Ênfase os Estudos na RPPN Fazenda Santa Clara (São João do Cariri)* (ed. Abílio, F. J. P.) (Editora UFPB, 2017).

39. Easdale, T. A., Gurvich, D. E., Sersic, A. N. & Healey, J. R. Tree morphology in seasonally dry montane forest in Argentina: Relationships with shade tolerance and nutrient shortage. *J. Veg. Sci.* **18**, 313–326 (2007).

40. Tomlinson, K. W. *et al.* Defence against vertebrate herbivores trades off into architectural and low nutrient strategies amongst savanna Fabaceae species. *Oikos* **125**, 126–136 (2016).

41. Cifuentes, D. A. G. Potential of Using Morphological and Functional Traits of Woody Species As Indicators of Dry Conditions in the Transition Zone of the Atlantic Forest (Mata Atlântica) in the Rio De Janeiro State, Brazil. (Universidad Autonoma de San Luis Potosí, 2018).

42. Silva, J. S. & Sales, M. F. de. O gênero Mimosa (Leguminosae-Mimosidae) na microrregião do Vale do Ipanema, Pernambuco. *Rodriguésia* **59**, 435–448 (2008).

43. Nurit, K., Agra, M. de F., Basílio, I. J. L. D. & Baracho, G. S. Flora da Paraíba, Brasil: Loganiaceae. *Acta Bot. Brasilica* **19**, 407–416 (2005).

44. Almeida, P. G. C. de, Souza, E. R. de & Queiroz, L. P. de. Flora da Bahia:

Leguminosae – Aliança Chloroleucon (Mimosoideae: Ingeae). *SITIENTIBUS série Ciências Biológicas* **15**, 1–22 (2015).

45. Asevedo, L., Winck, G. R., Mothé, D. & Avilla, L. S. Ancient diet of the Pleistocene gomphothere Notiomastodon platensis (Mammalia, Proboscidea, Gomphotheriidae) from lowland mid-latitudes of South America: Stereomicrowear and tooth calculus analyses combined. *Quat. Int.* **255**, 42–52 (2012).

46. Bargo, M. S. The ground sloth Megatherium americanum: Skull shape, bite forces, and diet. *Acta Palaeontol. Pol.* **46**, 173–192 (2001).

47. Bargo, M. S. & Vizcaíno, S. F. Paleobiology of Pleistocene ground sloths (Xenarthra, Tardigrada): Biomechanics, morphogeometry and ecomorphology applied to the masticatory apparatus. *Ameghiniana* **45**, 175–196 (2008).

48. Dantas, M. A. T. *et al.* Paleoecology and radiocarbon dating of the Pleistocene megafauna of the Brazilian Intertropical Region. *Quat. Res. (United States)* **79**, 61–65 (2013).

49. Dantas, M. A. T. & Cozzuol, M. A. The Brazilian Intertropical Fauna from 60 to About 10 ka B.P.: Taxonomy, Dating, Diet, and Paleoenvironments. in *Marine Isotope Stage 3 in Southern South America, 60 ka B.P. – 30 ka B.P.* (ed. Gasparini, G. M.) (Springer International Publishing, 2016).

50. Dantas, M. A. T. *et al.* Isotopic paleoecology (δ13C) of mesoherbivores from Late Pleistocene of Gruta da Marota, Andaraí, Bahia, Brazil. *Hist. Biol.* (2019) doi:10.1080/08912963.2019.1650742.

51. Davis, M. What north america's skeleton crew of megafauna tells us about

community disassembly. *Proc. R. Soc. B Biol. Sci.* **284**, 1–7 (2017).

52. De Melo França, L. *et al.* Chronology and ancient feeding ecology of two upper Pleistocene megamammals from the Brazilian Intertropical Region. *Quat. Sci. Rev.* **99**, 78–83 (2014).

53. Domingo, L., Prado, J. L. & Alberdi, M. T. The effect of paleoecology and paleobiogeography on stable isotopes of Quaternary mammals from South America. *Quat. Sci. Rev.* **55**, 103–113 (2012).

54. Feranec, R. S. Stable isotopes, hypsodonty, and the paleodiet of Hemiauchenia (Mammalia: Camelidae): A morphological specialization creating ecological generalization. *Paleobiology* **29**, 230–242 (2003).

55. Koch, P. L., Hoppe, K. A. & Webb, S. D. The isotopic ecology of late Pleistocene mammals in North America Part 1. Florida. *Chem. Geol.* **152**, 119–138 (1998).

56. de Oliveira, K. *et al.* Fantastic beasts and what they ate: Revealing feeding habits and ecological niche of late Quaternary Macraucheniidae from South America. *Quat. Sci. Rev.* **231**, (2020).

57. Owen-Smith, N. Contrasts in the large herbivore faunas of the southern continents in the late Pleistocene and the ecological implications for human origins. *J. Biogeogr.* **40**, 1215–1224 (2013).

58. Pansani, T. R., Muniz, F. P., Cherkinsky, A., Pacheco, M. L. A. F. & Dantas, M. A. T. Isotopic paleoecology (δ13C, δ18O) of Late Quaternary megafauna from Mato Grosso do Sul and Bahia States, Brazil. *Quat. Sci. Rev.* **221**, (2019).

59. Pereira, I. C. dos S., Dantas, M. A. T. & Ferreira, R. L. Record of the giant sloth

Valgipes bucklandi (Lund, 1839) (Tardigrada, Scelidotheriinae) in Rio Grande do Norte state, Brazil, with notes on taphonomy and paleoecology. *J. South Am. Earth Sci.* **43**, 42–45 (2013).

60. Ruez, D. R. Diet of Pleistocene Paramylodon harlani (Xenarthra: Mylodontidae): Review of methods and preliminary use of carbon isotopes. *Texas J. Sci.* **57**, 329–344 (2005).

61. Saarinen, J. & Karme, A. Tooth wear and diets of extant and fossil xenarthrans (Mammalia, Xenarthra) – Applying a new mesowear approach. *Palaeogeogr. Palaeoclimatol. Palaeoecol.* **476**, 42–54 (2017).

62. Smith, F. A. *et al.* Unraveling the consequences of the terminal Pleistocene megafauna extinction on mammal community assembly. *Ecography (Cop.).* **39**, 223–239 (2016).

63. Macfadden, B. J. *et al.* Ancient Feeding Ecology and Niche Differentiation of Pleistocene Mammalian Herbivores from Tarija , Bolivia : Morphological and Isotopic Evidence Published by : Cambridge University Press Stable URL : https://www.jstor.org/stable/2401158 REFERENCES Linked. **23**, 77–100 (1997).

64. Omena, É. C., Silva, J. L. L. da, Sial, A. N., Cherkinsky, A. & Dantas, M. A. T. Late Pleistocene meso-megaherbivores from Brazilian Intertropical Region: isotopic diet (δ 13C), niche differentiation, guilds and paleoenvironmental reconstruction (δ 13C, δ 18O). *Hist. Biol.* **00**, 1–6 (2020).

65. Sánchez, B., Prado, J. L. & Alberdi, M. T. Feeding ecology, dispersal, and extinction of South American Pleistocene gomphotheres (Gomphotheriidae, Proboscidea). *Paleobiology* **30**, 146–161 (2004).

66. Austin, D. F. The american Erycibeae (Convolvulaceae): *Maripa, Dicranostyles*, and *Lysiostyles* I. Systematics. *Annals of the Missouri Botanical Garden* **60**, 306-412 (1973).

67. Lewinsohn, T. M. The geographical distribution of plant latex. *Chemoecology* **2**, 64-68 (1991).

68. Martins, M. V. Leguminosas arbustivas e arbóreas de fragmentos florestais remanescentes no noroeste paulista, Brasil. (Universidade Estadual Paulista, 2009).

69. Martins, E. G. A. Leguminosas arbustivas e arbóreas de fragmentos florestais remanescentes no noroeste paulista, Brasil. (Universidade de São Paulo, 2009).

70. Moneiro, M. H. D. A., Neves, L. J., & Andreata, R. H. P. Taxonomia e anatomia das espécies de Pouteria aublet (Sapotaceae) do estado do Rio de Janeiro, Brasil. *Pesquisas Botânicas* **58**, 7-118 (2007).

71. Rudall, P. J. Laticifers in Euphorbiaceae-a conspectus. Journal of the Linnean Society **94**, 143-163 (1987).

72. Santos, C. C., Borba, E. L., & Queiroz, L. P. A família Anacardiaceae no semi-árido do estado da Bahia, Brasil. *Sitientibus Série Ciências Biologicas* **8**, 189-219 (2008).

73. Silva, L. A. A tribo Mimoseae bronn. (Leguminosae) no Espírito Santo. (Universidade Federal do Espírito Santo, 2016).

74. Kier, G. *et al.* Global patterns of plant diversity and floristic knowledge. J. Biogeogr. 32, 1107–1116 (2005).

75. Dinerstein, E. *et al.* An Ecoregion-Based Approach to Protecting Half the

Terrestrial Realm. *Bioscience* **67**, 534–545 (2017).

76. Engemann, K., Sandel, B., Boyle, B.L., Enquist, B.J., Jørgensen, P. M., Kattge, J., McGill B. J., Morueta-Holme, N., Peet, R.K., Spencer, N. J., Violle, C. , Wiser, S.K., Svenning, J.-C. A plant growth form dataset for the New World. *Ecology*, **97**, 3243-3243 (2016).

77. Govaerts, R. How Many Species of Seed Plants Are There? *Taxon*, **50**, 1085-1090 (2001).

78. Galetti, M. *et al.* Ecological and evolutionary legacy of megafauna extinctions. *Biol. Rev.* **93**, 845–862 (2018).

79. Charles-Dominique, T. *et al.* Spiny plants, mammal browsers, and the origin of African savannas. *Proc. Natl. Acad. Sci. U. S. A.* **113**, E5572–E5579 (2016).

80. Giacomo, M. D., & Fariña, R. A. Allometric models in paleoecology: Trophic relationships among Pleistocene mammals. *Palaeogeogr. Palaeoclimatol. Palaeoecol.* **471**, 15-30 (2017).

81. Hempson, G. P., Archibald, S. & Bond, W. J. A continent-wide assessment of the form and intensity of large mammal herbivory in Africa. *Science*. **350**, 1056–1061 (2015).

82. Absy, M.L. *et al.* Mise en évidence de quatre phases d'ouverture de la forêt dense dans le sud-est de l'Amazonie au cours des 60 000 dernières années. Première comparaison avec d'autres régions tropicales. *Comptes rendus de l'Académie des sciences* **312**, 673-678 (1991).

83. Behling, H. & Hooghiemstra, H. Environmental history of the Colombian savannas of the Llanos Orientales since the Last Glacial Maximum from lake records El Pinal and Carimagua. *J. Paleolim.* **21**, 461-476 (1999).

84. Behling, H. & Hooghiemstra, H. Holocene Amazon rainforest–savanna dynamics and climatic implications: high-resolution pollen record from Laguna Loma Linda in eastern Colombia. *J. Quatern. Sci.* **15**, 687–695 (2000).

85. Behling H & Lichte M. Evidence of dry and cold climatic conditions at glacial times in tropical Southeastern Brazil. *Quatern. Res.* **48**, 348-358 (1997).

86. Behling, H. & Negrelle, R.R.B. Tropical rain forest and climate dynamics of the atlantic lowland, southern Brazil, during the late quaternary. *Quatern. Res.* **56**, 383-389 (2001).

87. Behling, H. Late Quaternary vegetation, climate and fire history of the Araucaria forest and campos region from Serra Campos Gerais, Paraná State (South Brazil). *Rev Palaeobot. Palyno.* **97**, 109–121. (1997)

88. Behling, H. Late Quaternary vegetation, climate and fire history from the tropical mountain region of Morro de Itapeva, SE Brazil. *Palaeogeogr. Palaeoclimatol. Palaeoecol.* **129**, 407-422 (1997).

89. Behling, H. *et al.* Late Quaternary Araucaria Forest, grassland (Campos), fire and climate dynamics, studied by high-resolution pollen, charcoal and multivariate analysis of the Cambará do Sul core in southern Brazil. *Palaeogeogr. Palaeoclimatol. Palaeoecol.* **203**, 277-297. (2004)

90. De Freitas, H. A. *et al.* Late Quaternary vegetation dynamics in the southern Amazon Basin inferred from carbon isotopes in soil organic matter. *Quatern. Res.* **55**, 39–46 (2001).

91. De Oliveira, P.E. *et al.* Late Pleistocene/Holocene climatic and vegetational history of the Brazilian caatinga: the fossil dunes of the middle Sao Francisco River. *Palaeogeogr. Palaeoclimatol. Palaeoecol.* **152**, 319-337 (1999).

92. Garcia, M.J. *et al.* A Holocene vegetational and climatic record from the Atlantic rainforest belt of coastal State of São Paulo, SE Brazil. *Rev Palaeobot. Palyno.* **131**, 181–99. (2004).

93. Mayle, F.E. & Power, M.J. Impact of a drier Early–Mid-Holocene climate upon Amazonian forests. *Phil. Trans. R. Soc. B,* **363**, 1829-1838 (2008).

94. Mayle, F. E., Burbridge, R. & Killeen, T. J. Millennial-scale dynamics of southern Amazonian rain forests. *Science* **290**, 2291–2294 (2000).

95. Burbridge, R. E., Mayle, F. E. & Killeen, T. J. Fifty-thousand-year vegetation and climate history of Noel Kempff Mercado National Park, Bolivian Amazon. *Quatern. Res.* **61**, 215-230 (2004).

96. Parizzi, M. G. *et al.* Genesis and Environmental History of Lagoa Santa, Southeastern Brazil. *The Holocene,* 8, 311-321 (1998).

97. Salgado-Labouriau, M. L. *et al.* A dry climatic event during the late Quaternary of tropical Brazil. *Rev Palaeobot. Palyno.* **99**, 115-129 (1998).

98. Sifeddine, A. et al. (2003) A 21 000 cal years paleoclimatic record from Caçó Lake, northern Brazil: evidence from sedimentary and pollen analyses. *Palaeogeogr. Palaeoclimatol. Palaeoecol.* **189**, 25–34.

99. Van der Hammen, T. & Absy, M. Amazonia during the last glacial *Palaeogeogr. Palaeoclimatol. Palaeoecol.* **109**, 247-261 (1994).

100. Veríssimo, N. *et al.* Holocene vegetation and fire history of the Serra do Caparaó, SE Brazil. *The Holocene*, **22**, 1243-1250 (2012).

101. Behling, H. South and southeast Brazilian grasslands during Late Quaternary times: a synthesis. *Palaeogeogr. Palaeoclimatol. Palaeoecol* **177**, 19–27 (2002).
