# Supplementary material for: The legacy of the extinct Neotropical megafauna on plants and biomes
Source: Nat Commun. 2022 Jan 10;13:129. doi: 10.1038/s41467-021-27749-9 (PMC8748933; doi:10.1038/s41467-021-27749-9)
Supplement: Supplementary file 2 — Reporting Summary [file 41467_2021_27749_MOESM2_ESM.pdf]

nature research

Corresponding author(s): Vinícius de Lima Dantas

Last updated by author(s): Jul 1, 2021

# Reporting Summary

Nature Research wishes to improve the reproducibility of the work that we publish. This form provides structure for consistency and transparency in reporting. For further information on Nature Research policies, see our Editorial Policies and the Editorial Policy Checklist.

## Statistics

For all statistical analyses, confirm that the following items are present in the figure legend, table legend, main text, or Methods section.

| n/a | Confirmed | |
|---|---|---|
| ☐ | ☒ | The exact sample size (*n*) for each experimental group/condition, given as a discrete number and unit of measurement |
| ☐ | ☒ | A statement on whether measurements were taken from distinct samples or whether the same sample was measured repeatedly |
| ☐ | ☒ | The statistical test(s) used AND whether they are one- or two-sided<br>*Only common tests should be described solely by name; describe more complex techniques in the Methods section.* |
| ☐ | ☒ | A description of all covariates tested |
| ☐ | ☒ | A description of any assumptions or corrections, such as tests of normality and adjustment for multiple comparisons |
| ☐ | ☒ | A full description of the statistical parameters including central tendency (e.g. means) or other basic estimates (e.g. regression coefficient) AND variation (e.g. standard deviation) or associated estimates of uncertainty (e.g. confidence intervals) |
| ☐ | ☒ | For null hypothesis testing, the test statistic (e.g. *F*, *t*, *r*) with confidence intervals, effect sizes, degrees of freedom and *P* value noted<br>*Give P values as exact values whenever suitable.* |
| ☐ | ☒ | For Bayesian analysis, information on the choice of priors and Markov chain Monte Carlo settings |
| ☐ | ☒ | For hierarchical and complex designs, identification of the appropriate level for tests and full reporting of outcomes |
| ☐ | ☒ | Estimates of effect sizes (e.g. Cohen's *d*, Pearson's *r*), indicating how they were calculated |

*Our web collection on statistics for biologists contains articles on many of the points above.*

## Software and code

Policy information about availability of computer code

| | |
|---|---|
| Data collection | The raw data used in this study was downloaded from published datasets in internet repositories, including Remote Sensing and plant trait databases, or compiled from published articles. The resulting spreadsheets were imported and processed in R v. 4.0.2, using the packages "raster" (v. 3.4-5), "rgbif" (v. 3.5.2), "sp" (v. 1.4-2), "rgdal" (v. 1.5-12), "latticeExtra" (v. 0.6-29),"lattice" (v. 0.20-41), and "rgeos" (v. 0.5-5). This processing involved filtering, merging and aggregating plant trait, megafauna, environmental and disturbance data to the ecoregion (the scale at which analyses were carried) before statistical analyses, calculating means across individual observations for the ecoregion's polygons and mapping relevant variables. Custom codes were deposited in Zenodo (DOI: 10.5281/zenodo.5752131). |
| Data analysis | Data analysis was also performed in R v. 4.0.2, using the base package, as well as "olsrr" (v. 0.5.3),"parameters" (v. 0.13.0), "spatialreg" (v. 1.1-5), "sandwich" (v. 3.0-0), "raster" (v. 3.4-5), "lmtest" (v. 0.9-37), "dominanceanalysis" (v. 2.0.0), "spdep" (v. 1.1-7),"sp" (v. 1.4-2), "FSA" (v. 0.8.32) "effectsize" (v. 0.4.4-1), "vegan" (v. 2.5-7), and "pscl"(v. 1.5.5). The analyses included general and generalized (binomial family) linear models, checking of model assumptions (e.g. spatial autocorrelation in the residuals), principal component analysis, hierarchical clustering and comparisons of antiherbiomes using Kruskal-Wallis and Dunn tests. Custom codes were deposited in Zenodo (DOI: 10.5281/zenodo.5752131). |

For manuscripts utilizing custom algorithms or software that are central to the research but not yet described in published literature, software must be made available to editors and reviewers. We strongly encourage code deposition in a community repository (e.g. GitHub). See the Nature Research guidelines for submitting code & software for further information.

## Data

Policy information about availability of data

All manuscripts must include a data availability statement. This statement should provide the following information, where applicable:

- Accession codes, unique identifiers, or web links for publicly available datasets
- A list of figures that have associated raw data
- A description of any restrictions on data availability

This study is based on open source data compiled from the literature or downloaded published datasets, such as: MegaPast2Future/PHYLACINE_1.2 (http://doi.org/10.5281/zenodo.3690867), Diet preferences in terrestrial mammals worldwide (https://doi.org/10.5061/dryad.6cd0v), WorldClim2 (http://www.worldclim.com/version2), SoilGrids (https://www.isric.org/explore/soilgrids), MODIS active fire location product (https://modis-fire.umd.edu/af.html), HURDAT2 (https://www.nhc.noaa.gov/data/#hurdat), Global wood density database (doi:http://hdl.handle.net/10255/dryad.235), PalmTraits 1.0 (doi:https://doi.org/10.5061/dryad.ts45225), Dantas and Pausas, 2020 (https://doi.org/10.5061/dryad.3xsj3txc0), Flora do Brasil (http://floradobrasil.jbrj.gov.br) , GBIF (www.gbif.org) and Ecoregions2017 (https://ecoregions.appspot.com/). The curated data generated in this study have been deposited in the Zenodo database (DOI: 10.5281/zenodo.5752131) and was also added as Supplementary Data alongside this article. A Source Data table containing the PCA scores used for antiherbiome contrasts is also provided alongside this article.

# Field-specific reporting

Please select the one below that is the best fit for your research. If you are not sure, read the appropriate sections before making your selection.

☐ Life sciences ☐ Behavioural & social sciences ☒ Ecological, evolutionary & environmental sciences

For a reference copy of the document with all sections, see nature.com/documents/nr-reporting-summary-flat.pdf

# Ecological, evolutionary & environmental sciences study design

All studies must disclose on these points even when the disclosure is negative.

| | |
|---|---|
| Study description | In this study, we tested three hypotheses: 1) That geographic patterns in plant defences against herbivory in the Neotropics are explained by extinct megafauna historical distribution; 2) That the resulting trait patterns allow the recognition of antiherbiomes (large regions characterized by convergent plant defence strategies) similar to those recognized for the megafauna-rich Afrotropical region; 3) That some of the current Neotropical forest ecoregions used to be savanna-dominated during the Pleistocene. For this, we compiled species-level data from literature sources on plant defences for Neotropical woody species, merged these with distribution data for these same species, and calculated ecoregion level trait values for the entire plant assemblages within each Neotropical ecoregions. We also obtained data on extinct megafauna and current mammal herbivore distribution, body mass and diet (grazing, browser and mixed-feeders), from which we were able to calculate mean richness, body mass, as well as the specific richness according to diet type. We also obtained and calculated ecoregion level means for environmental (climate, soil) and disturbance (fire count and intensity, and hurricane count) data from global maps, and visually determined insularity using ecoregion maps (classifying ecoregions as either continental or insular). These variables were used to predict both, megafauna indicators and traits (here, specifically including megafauna and extant herbivore indicators among the predictors), in order to answer the first question. Model simplification was carried using AIC, and the average explained variance was calculated for each selected variable using R2 or Pseudo-R2 (depending on response variable type). The exact number of observations in this statistical test depended on the amount of continental (islands were excluded based on evidence of insularities effect that could mask the effect of megafauna) ecoregions with available trait, environment and disturbance data, and ranged from 131 (leaf spines) to 142 (the other three traits) out of 150 continental ecoregions (island ecoregions were not considered, as explained in "Data Exclusions" below). The second question was tested using a principal component analysis on the ecoregion-by-trait matrix, followed by hierarchical clustering. This analysis was performed with all 179 ecoregions. Missing values were completed using the means, except for leaf spines, in which we used predicted values due to the higher number of missing values (20 against less than 7 for the other traits). We also compared climate, soil, disturbance and megafauna differences among insular ecoregions using Kruskal-Wallis and post-hoc Dunn tests. The number of observations was 150 for the megafauna indicators, 148 for climate variables, 146 for soil variables and fire frequency, and 143 for fire intensity. The third question was answered by subsetting ecoregions of antiherbiomes characterized by highly defended species, in which the vegetation is currently forest, and that used to harbour a very rich megafauna, including many now extinct large grazers (more or equal to the 75% quantile, in both cases). We then compared their locations with that of fossil sites presenting evidence suggestive of a savanna-dominated state in the Pleistocene. This process involved evaluating antiherbiome classification, current vegetation and megafauna indicators for all the 179 ecoregions. |
| Research sample | We used all Neotropical ecoregions with available data (179). The Neotropical region was selected because it is a region that was greatly affected by megafauna extinction (had numerous extinct megafauna species), for which little is known on the consequences of megafauna history on vegetation patterns, and for which there is sufficient trait data available in the literature. The use of ecoregion as observation unit results from the fact that ecoregions are regionalized based on species composition, providing an appropriate framework for merging species-level trait and distribution data, thus, overcoming the current scarcity of field studies recording plant traits. This is especially possible because, for most plant traits, interspecific variability is much greater than intraspecific variability. Our trait data is for woody plants, here interpreted as including palms. This data was collected as a sample of the dominant species in the Neotropical woody flora and inclydes all available data. The compiled data came from published scientific articles, as well as published datasets derived from scientific articles and remote sensing products (e.g. MODIS fire products). It also includes data from Flora databases available from the internet, associated with Botanic Gardens, such as the "Flora do Brasil" database. Finally, it includes distribution data provided by the Global Biodiversity Information Facility (GBIF). |
| Sampling strategy | The sampling was carried by making searches in Google Scholar looking for global datasets on species-level plant defence traits, |

megafauna and herbivore historical distribution, climate, soil, fire and hurricane datasets. When a compiled global dataset could not be found (i.e. for stem spines), species level data was collected from numerous sources by making searches in Google Scholar using key words and including all the available data. Thus, the sample size was determined by the availability of trait, megafauna, climate, soil, fire and hurricane data for the 179 Neotropical ecoregions. Considering that the number of predictor variables was never higher than 15 and that the number of observations was never smaller than 131, the later was sufficiently large to avoid problems associated with degrees of freedom.

| | |
|---|---|
| Data collection | The data was collected by me during the first semester of 2020 as described in previous boxes. The trait data was collated in a Excel spreadsheet and inserted in R as .csv or .txt files. In some cases, the file provided in the dataset was directly used. Distribution data for plant species was downloaded from GBIF using the rgbif package and codes supplied by the GBIF helpdesk. Trait and herbivore distributions, as well as fire (MODIS active fire location product; MCD14ML) and hurricame (HURDAT2) data comming from large datasets were generally downloaded as csv or txt files and imported to R. Climate and soil data were downloaded as .geotiff files and imported to R, after which data was extracted using the Ecoregions shapefile. The shapefile containing Ecoregion limits was also imported to R, and was obtained from the internet from the Ecoregions2017 webpage. Links to these datasets are provided in "Data" |
| Timing and spatial scale | All data was collected in 2020. The data started to be collected in May 25th, and finished in July 7th. After a first submission to another journal (and the editor's final decision), we followed a reviewer's suggestion and collected additional data on megafauna diet, which started in September 9th and finished in October 30th. Data comes from field studies and therefore, have variable spatial resolution and were collected at variable periods of time. The extent of the study is the Neotropical biogeographic region. |
| Data exclusions | As explained in the manuscript, we specifically excluded data from insular ecoregions for statistical tests involving trait-megafauna relationships. This was done based on our own results (described in the main text and shown in the supplementary material) supporting our initial hypothesis that, as predicted by the island biogeography theory, megafauna species richness in island would be reduced, consistent with lower colonization and higher extinction rates in islands. Therefore, in islands, megafauna richness was not an appropriate proxy for animal abundance, and was not be used to evaluate the relationship between herbivore abundance and plant defences. Therefore, the exclusion criteria was pre-established based on theory and confirmed based on our own results showing an effect of insulatity on megafauna patterns, matching expectations from theory. |
| Reproducibility | This is not an experimental study and all the measurements were performed by the authors of the primary research papers following well established protocols. The data used in this study was compiled using keywords and free web-based search engines (Google Scholar). |
| Randomization | Do not apply as this is an observational study. |
| Blinding | Do not apply as this is an observational study. |

Did the study involve field work? ☐ Yes ☒ No

# Reporting for specific materials, systems and methods

We require information from authors about some types of materials, experimental systems and methods used in many studies. Here, indicate whether each material, system or method listed is relevant to your study. If you are not sure if a list item applies to your research, read the appropriate section before selecting a response.

## Materials & experimental systems

| n/a | Involved in the study | |
|---|---|---|
| ☒ | ☐ | Antibodies |
| ☒ | ☐ | Eukaryotic cell lines |
| ☒ | ☐ | Palaeontology and archaeology |
| ☒ | ☐ | Animals and other organisms |
| ☒ | ☐ | Human research participants |
| ☒ | ☐ | Clinical data |
| ☒ | ☐ | Dual use research of concern |

## Methods

| n/a | Involved in the study | |
|---|---|---|
| ☒ | ☐ | ChIP-seq |
| ☒ | ☐ | Flow cytometry |
| ☒ | ☐ | MRI-based neuroimaging |
