# Supplementary material for: The legacy of the extinct Neotropical megafauna on plants and biomes
Source: Nat Commun. 2022 Jan 10;13:129. doi: 10.1038/s41467-021-27749-9 (PMC8748933; doi:10.1038/s41467-021-27749-9)
Supplement: Supplementary file 4 — Description of Additional Supplementary Files [file 41467_2021_27749_MOESM4_ESM.pdf]

Title: Supplementary Data 1

Description: Dataset containing (1) ecoregion- and species- level data on five plant functional traits (wood density, leaf size, stem spines, leaf spines and latex production); (2) ecoregion-level data on extinct megafauna historical patterns, fire, climate, soil, hurricanes and insularity; (3) plant, extinct megafauna and extant mammal herbivore species occurrences per ecoregion; and (4) diet information for megafauna species.
